# CNS erythroblastic sarcoma: a potential emerging pediatric tumor type characterized by *NFIA::RUNX1T1/3* fusions

**DOI:** 10.1186/s40478-023-01708-5

**Published:** 2024-01-19

**Authors:** Arnault Tauziède-Espariat, Lucille Lew-Derivry, Samuel Abbou, Alice Métais, Gaëlle Pierron, Stéphanie Reynaud, Julien Masliah-Planchon, Cassandra Mariet, Lauren Hasty, Volodia Dangouloff-Ros, Nathalie Boddaert, Marie Csanyi, Aude Aline-Fardin, Claire Lamaison, Fabrice Chrétien, Kévin Beccaria, Stéphanie Puget, Pascale Varlet

**Affiliations:** 1 Department of Neuropathology, GHU Paris-Psychiatrie et Neurosciences, Sainte-Anne Hospital, 1, rue Cabanis, 75014 Paris, France; 2 Inserm, UMR 1266, IMA-Brain, Institut de Psychiatrie et Neurosciences de Paris, Paris, France; 3 Department of Hematology and Pediatric Oncology, Armand Trousseau Hospital, Paris, France; 4 Children and Adolescent Oncology Department, INSERM U1015, Paris-Saclay University, Villejuif, France; 5 Paris-Sciences-Lettres, Curie Institute Research Center, INSERMU830, Paris, France; 6 Laboratory of Somatic Genetics, Curie Institute Hospital, Paris, France; 7 Pediatric Radiology Department, Hôpital Necker Enfants Malades, AP-HP, Paris, France; 8 UMR 1163, Institut Imagine and INSERM U1299, Université Paris Cité, Paris, France; 9 Institute of Pathology, Centre de Biologie Pathologie, Lille University Hospital, 59000 Lille, France; 10 Department of Pathology, CHU de La Martinique, Fort-de-France, France; 11 Department of Pathology, Henri Mondor Hospital, APHP, Créteil, France; 12 Department of Pediatric Neurosurgery, Necker Hospital, APHP, Université Paris Descartes, Sorbonne Paris Cite, 75015 Paris, France; 13 Department of Pediatric Neurosurgery, CHU de La Martinique, Fort-de-France, France

**Keywords:** Myeloid sarcoma, NFIA::RUNX1T1, Central nervous system, CNS leukemia

## Abstract

Erythroblastic sarcoma (ES) (previously called chloroma or granulocytic sarcoma) are rare hematological neoplams characterized by the proliferation of myeloid blasts at extramedullary sites, and primarily involve the skin and soft tissue of middle-aged adults. ES may be concomitant with or secondary to myeloid neoplasms (mostly acute myeloid leukemia (AML)) or in isolated cases (de novo) without infiltration of the bone marrow by blasts. ES share cytogenetic and molecular abnormalities with AML, including *RUNX1T1* fusions. Some of these alterations seem to be correlated with particular sites of involvement. Herein, we report an isolated erythroblastic sarcoma with *NFIA::RUNX1T1* located in the central nervous system (CNS) of a 3-year-old boy. Recently, two pediatric cases of CNS MS with complete molecular characterization have been documented. Like the current case, they concerned infants (2 and 3 years-old) presenting a brain tumor (pineal involvement) with leptomeningeal dissemination. Both cases also harbored a *NFIA::RUNX1T3* fusion. ES constitutes a diagnostic challenge for neuropathologists because it does not express differentiation markers such as CD45, and may express CD99 which could be confused with CNS Ewing sarcoma. CD43 is the earliest pan-hematopoietic marker and CD45 is not expressed by erythroid lineage cells. E-cadherin (also a marker of erythroid precursors) and CD117 (expressed on the surface of erythroid lineage cells) constitute other immunhistochemical hallmarks of ES. The prognosis of patients with ES is similar to that of other patients with AML but de novo forms seem to have a poorer prognosis, like the current case. To conclude, pediatric ES with *NFIA::RUNX1T1/3* fusions seem to have a tropism for the CNS and thus constitute a potential pitfall for neuropathologists. Due to the absence of circulating blasts and a DNA-methylation signature, the diagnosis must currently be made by highlighting the translocation and expression of erythroid markers.

## Introduction

Erythroblastic sarcoma (ES) (previously called chloroma or granulocytic sarcoma) are rare hematological neoplams characterized by the proliferation of myeloid blasts at extramedullary sites, and primarily involve the skin and soft tissue of middle-aged adults [1]. ES may be concomitant with or secondary to myeloid neoplasms (mostly acute myeloid leukemia (AML)) or in isolated cases (de novo) without infiltration of the bone marrow by blasts [1]. ES share cytogenetic and molecular abnormalities with AML, including *RUNX1T1* fusions [1, 2]. Some of these alterations seem to be correlated with particular sites of involvement (such as *RUNX1::RUNX1T1* fusions for pediatric orbital tumors) [1].

## Case presentation

**Fig. 1:**
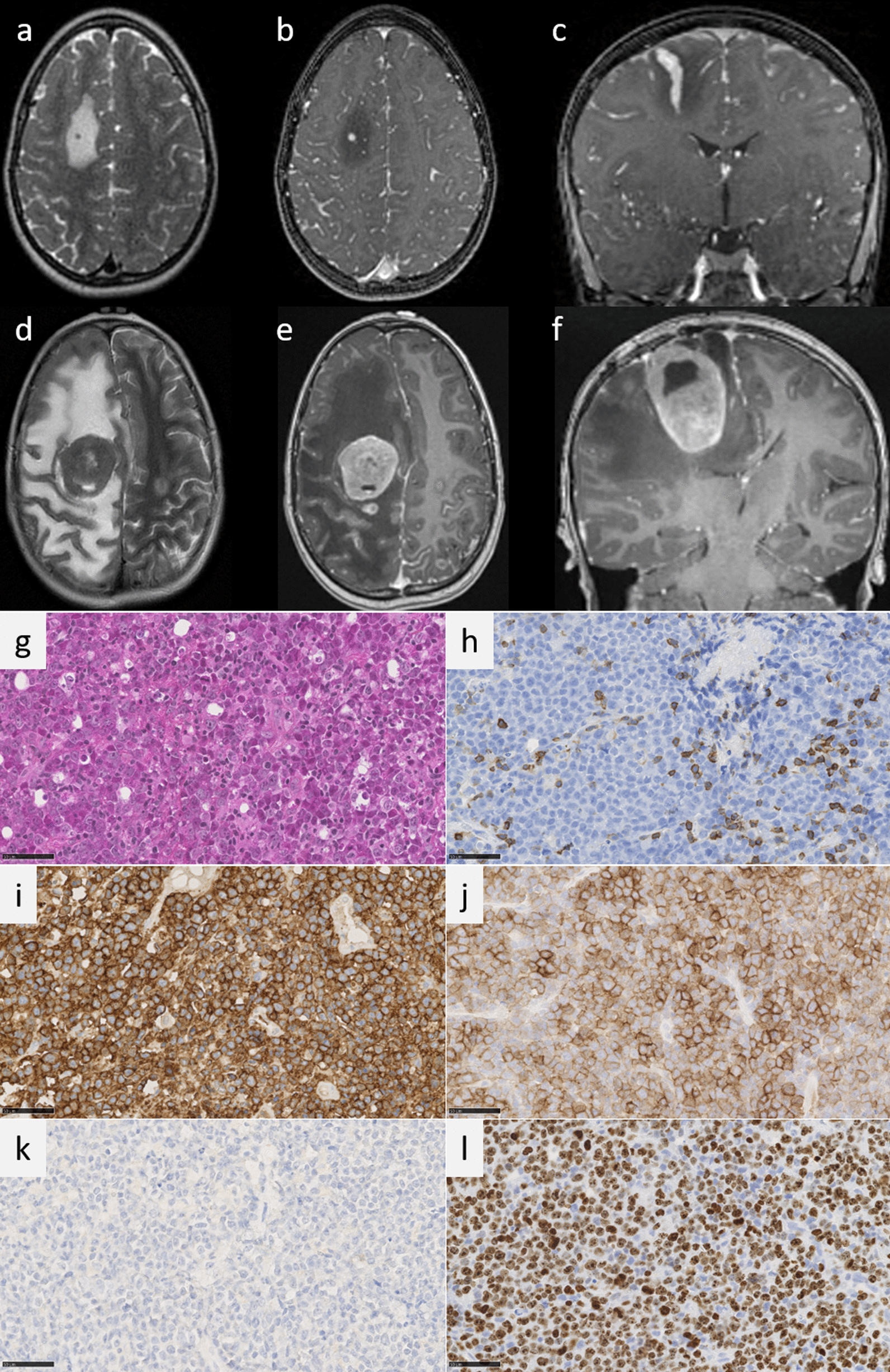
Radiological and histopathological features the case Axial T2-weighted (**a**, **d**), axial (**b**, **e**) and coronal (**c**, **f**) T1-weighted images after gadolinium injection **a**, **b**, **c**: initial MRI, showing a thick linear enhancement within the right frontal lobe, and important peritumoral edema (high-T2-weighted signal), associated with thick and diffuse leptomeningeal enhancement. Frontal tumor enhancement showed restricted diffusion (image not shown). **d**, **e**, **f**: The follow-up MRI, 3 months after partial surgery, showing an increased solid tumor volume and larger peritumoral edema. The biopsy highlighted a dense proliferation composed of sheets of undifferentiated cells with numerous mitotic figures (**g** HPS, magnification × 400). Tumor cells were immunonegative for CD45 which highlighted some normal lymphocytes (**h** magnification × 400). Diffuse expression of CD43 (**i** magnification × 400) and CD117 (**j** magnification × 400). No immunoexpression for E-cadherin (**k** magnification 400x). High MIB1 labeling index (**l** magnification × 400). Scale bars represent 50 µm. HPS: Hematoxylin Phloxin Saffron

**Fig. 2:**
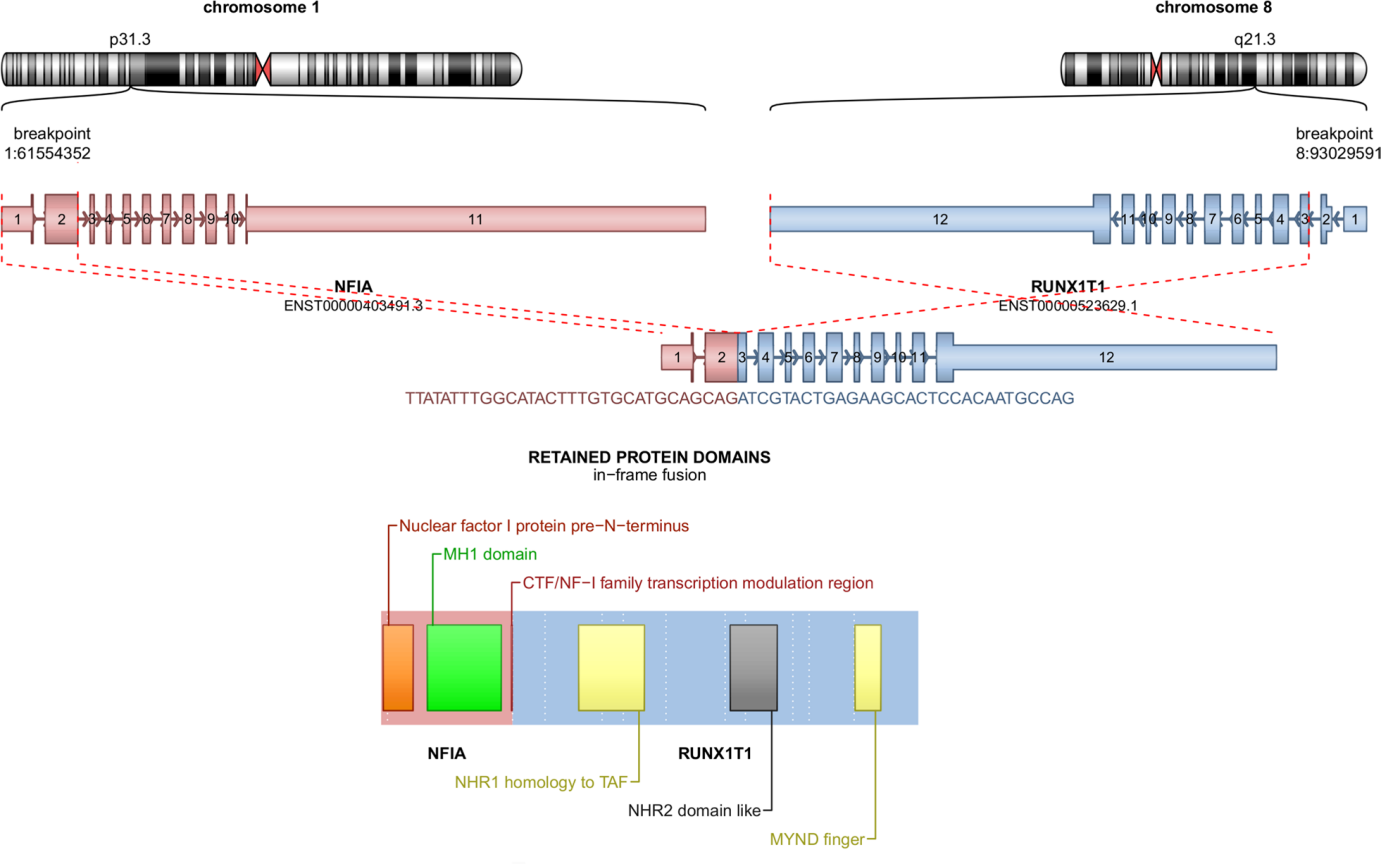
Genetic features RNAseq analysis highlights a fusion between *NFIA* (pink) and *RUNX1T1* (blue) genes, respectively located on chr1p31.3 and chr8q21.3

Herein, we report an isolated erythroblastic sarcoma located in the central nervous system (CNS) of a previously healthy 3-year-old boy, who suddenly presented with epileptic seizures and post-critic left hemiplegia. Magnetic resonance imaging (MRI) revealed a right frontal lesion associated with leptomeningeal dissemination (Fig. 1a-c). A biopsy of the lesion showed an undifferentiated proliferation composed of sheets of large cells with hyperchromatic nuclei, prominent nucleoli, brisk mitotic activity and apoptotic bodies (Fig. 1d). INI1 and BRG1 stainings were maintained and there was no expression of Lin28A, CD34, glial (GFAP and Olig2), neuronal (MAP2, NeuN, synaptophysin), melanocytic (SOX10, HMB45), myogenic (desmin and myogenin), or lymphoid (CD45, CD3 and CD20) markers (Fig. 1e). The MIB1 labeling index was greater than 90% (Fig. 1f). There was no immunoreactivity for BCOR, NUT, CD99, or ETV4. DNA-methylation analysis was unable to classify the tumor. A cytological study of the cerebrospinal fluid (CSF) showed 300 tumoral cells/mm^3^. The complete blood count was normal and the bone marrow failed to reveal any blastic proliferation. Because the RNA-sequencing analysis revealed the presence of a *NFIA::RUNX1T1* fusion (Fig. 2), a final diagnosis of ES was suggested. Complementary immunohistochemical analyses showed the expression of CD117 and CD43, but no immunopositivity for E-cadherin was observed (Fig. 1g–i). The patient presented a neurological impairment with decreased consciousness and therefore received steroids and a first line of empiric chemotherapy adapted to sarcoma, based on the first histological results before the RNA sequencing was available (vincristin, doxorubicin, cyclophosphamide, and then etoposide and ifosfamide) [3]. This treatment was rapidly efficient for the consciousness disorders. When the final diagnosis was available, the treatment was adapted and the patient was treated in accordance with the Myechild 01 Trial with mitoxantrone (12 mg/m^2^ × 3) and cytarabine (100 mg/m^2^ × 4). Because of the tumor’s rapid local progression (Fig. 1j–l), and symptoms of intracranial hypertension (IH), the treatment was intensified with higher dose of cytarabine (12 g/m^2^ total dose) but was inefficient. Intratechal (IT) chemotherapy injections were not possible due to the IH. A subtotal resection was performed, and an Ommaya reservoir was put into place for intratechal injections of cytarabine, methotrexate and steroids. Despite 7 IT injections and a high intravenous dose of Methorexate associated with Erwinase injections, the CSF was still blastic and the subsequent progressive disease led to a rapid decline. The patient expired three months after symptoms began.

## Discussion and conclusions

Acute erythroid leukemias (AELs) represent only 1.2% of pediatric AML [4]*.* CNS localizations of ES are exceptionally rare in the literature, with a subset of them being secondary locations of myeloid neoplasms (AML) [2]. Recently, two pediatric cases of CNS ES with complete molecular characterization have been documented [5, 6]. Like the current case, they concerned infants (2 and 3 years-old) presenting a brain tumor (pineal involvement) with leptomeningeal dissemination [5, 6]. Both cases also harbored a *NFIA::RUNX1T3* fusion [5, 6], whereas the current case presented a *NFIA::RUNX1T1* fusion, also previously described in one pediatric abdominal ES case [7]. *NFIA* belongs to the NF1 family of transcription factors which is required for erythroid differentiation. *RUNX1T1* (also named as *CBFA2T1*) and *RUNX1T3* (also known as *CBFA2T3*) are part of the MTG (Myeloid Transcription Genes) family of transcriptional regulators which repress gene transcription. ES constitutes a diagnostic challenge for neuropathologists because it does not express differentiation markers such as CD45, and may express CD99 which could be confused with CNS Ewing sarcoma [5, 6]. An extensive immunohistochemical analysis allows to eliminate high-grade gliomas, embryonal tumors (particularly atypical teratoid and rhabdoid tumor), sarcomas, *CIC-*rearranged, and primary rhabdomyosarcomas. CD43 is the earliest pan-hematopoietic marker and CD45 is not expressed by erythroid lineage cells [8]. E-cadherin (also a marker of erythroid precursors) and CD117 (expressed on the surface of erythroid lineage cells) constitute other immunhistochemical hallmarks of ES [5–7, 9]. The prognosis of patients with de novo ES seems to be pejorative but further reports are needed to conclude [1, 6].

To conclude, pediatric ES with *NFIA::RUNX1T1/3* fusions seem to have a tropism for the CNS and thus constitute a potential pitfall for neuropathologists. Due to the absence of circulating blasts and a DNA-methylation signature, the diagnosis must currently be made by highlighting the translocation and expression of erythroid markers.
